# Hepatic steatosis mediates the relationship between cholecystectomy and BMI increase: a population-based study

**DOI:** 10.3389/fmed.2025.1620036

**Published:** 2025-09-30

**Authors:** Ji Cao, Wenjun Mao, Qian Jiang, Xiaowei Huang, Yunyan Tan, Fei Zuo, Tianping Luo

**Affiliations:** Department of General Surgery, Changzhou Traditional Chinese Medicine Hospital, Affiliated Hospital of Nanjing University of Chinese Medicine, Changzhou, China

**Keywords:** cholecystectomy, body mass index, hepatic steatosis, mediation analysis, NHANES

## Abstract

**Background:**

Cholecystectomy has been linked with adverse metabolic outcomes, but its specific association with body mass index (BMI) and the underlying mechanisms remain insufficiently understood. This study aimed to assess the impact of cholecystectomy on BMI and examine the mediating role of hepatic steatosis.

**Methods:**

A total of 7,452 adults were included in this cross-sectional analysis. Baseline demographic, clinical, and laboratory data were compared between participants with and without cholecystectomy. Multivariable linear regression was used to evaluate the relationship between cholecystectomy and BMI, with progressive adjustment for demographic, clinical, and metabolic-inflammatory confounders. Subgroup and stratified analyses, propensity score modeling, and causal mediation analysis were conducted to validate findings and elucidate potential mechanisms.

**Results:**

Participants with cholecystectomy were older (mean age 61.0 vs. 49.5 years), more likely to be female, and exhibited higher BMI (mean 31.8 ± 8.1 cholecystectomy group vs. 29.6 ± 7.3 non-cholecystectomy, *p* < 0.05) as well as increased prevalence of hypertension, diabetes, and markers of inflammation (all *p* < 0.05). Cholecystectomy was independently associated with higher BMI after adjustment for confounders (fully adjusted β = 0.84 kg/m^2^; 95% CI: 0.09–1.59; *p* = 0.027), a finding robust to propensity score methods (overlap weighting β = 0.91; 95% CI: 0.55–1.27; *p* < 0.001). Stratified analyses indicated a more pronounced BMI increase among younger patients ( ≤ 50 years, β = 2.3; 95% CI: 0.83–3.76; *p* = 0.002), with no significant difference observed across postoperative time intervals. Causal mediation analysis demonstrated that hepatic steatosis, quantified by controlled attenuation parameter (CAP), mediated approximately 46% of the association between cholecystectomy and increased BMI (ACME = 0.806 kg/m^2^, 95% CI: 0.321–1.314; *p* = 0.002), while a significant direct effect of cholecystectomy remained (ADE = 0.964, 95% CI: 0.131–1.801) in bootstrap validation.

**Conclusions:**

Cholecystectomy is independently associated with increased BMI, particularly among younger adults, and this relationship is partially mediated by hepatic steatosis. These findings highlight the need for long-term metabolic monitoring and targeted interventions in patients undergoing cholecystectomy.

## Introduction

The advent of laparoscopic cholecystectomy in the late 1980s fundamentally transformed the management of gallstone disease (1). By the early 21st century, due to its minimally invasive nature and favorable safety profile—including low rates of mortality and morbidity—this procedure had become the standard therapeutic approach (2). Nonetheless, emerging evidence indicates that cholecystectomy is not without long-term consequences. Postoperative sequelae have attracted increasing scrutiny, with complications such as diarrhea—affecting approximately 13.3% of patients (3)—raising questions about possible postoperative changes in body weight, including both weight loss and weight gain. Most patients exhibit minimal fluctuations immediately after surgery, yet accumulating data indicate a gradual increase in body weight over time, particularly among individuals who receive dietary counseling 6 months postoperatively (4). In parallel, increasing attention has been paid to the role of the gallbladder and bile acids in systemic metabolic regulation. Notably, cholecystectomy affects bile acid storage and excretion, which may influence metabolic processes and lead to conditions like metabolic dysfunction-associated steatotic liver disease and metabolic syndrome (5). Consistent findings in animal models demonstrate increased hepatic fat accumulation following gallbladder removal (6). Furthermore, cholecystectomy has been linked to a higher risk of liver diseases, including nonalcoholic fatty liver disease, cirrhosis, and primary liver cancer (7). Despite these advances, the long-term association between cholecystectomy and changes in body mass index (BMI) remains inadequately characterized, and the mediating role of hepatic steatosis—precisely measured by controlled attenuation parameter (CAP)—in driving weight gain is undefined. To address this knowledge gap, the present population-based study investigates the association between cholecystectomy and BMI, with particular focus on the mediating effect of fatty liver disease in the long-term metabolic consequences of gallbladder removal.

## Method

### Research design

The National Health and Nutrition Examination Survey (NHANES) is a comprehensive, nationwide program that collects representative health data from both adults and children in the United States through interviews, physical examinations, and laboratory tests. The survey protocol was approved by the National Center for Health Statistics (NCHS) review board, and all participants provided informed consent. Data collection occurs biennially. For the purposes of this study, we utilized NHANES data from 2017 to 2020, as gallstone-related questionnaire responses were available only during this period. Although subsequent cycles (2021–2023) included questions on gallstones and cholecystectomy history, crucial information such as liver function tests and specific dates of surgery was not collected, precluding their inclusion in our analysis. Further information about the NHANES program and access to its data can be obtained from the official website (https://www.cdc.gov/nchs/nhanes/).

### Study population

**Figure 1:**
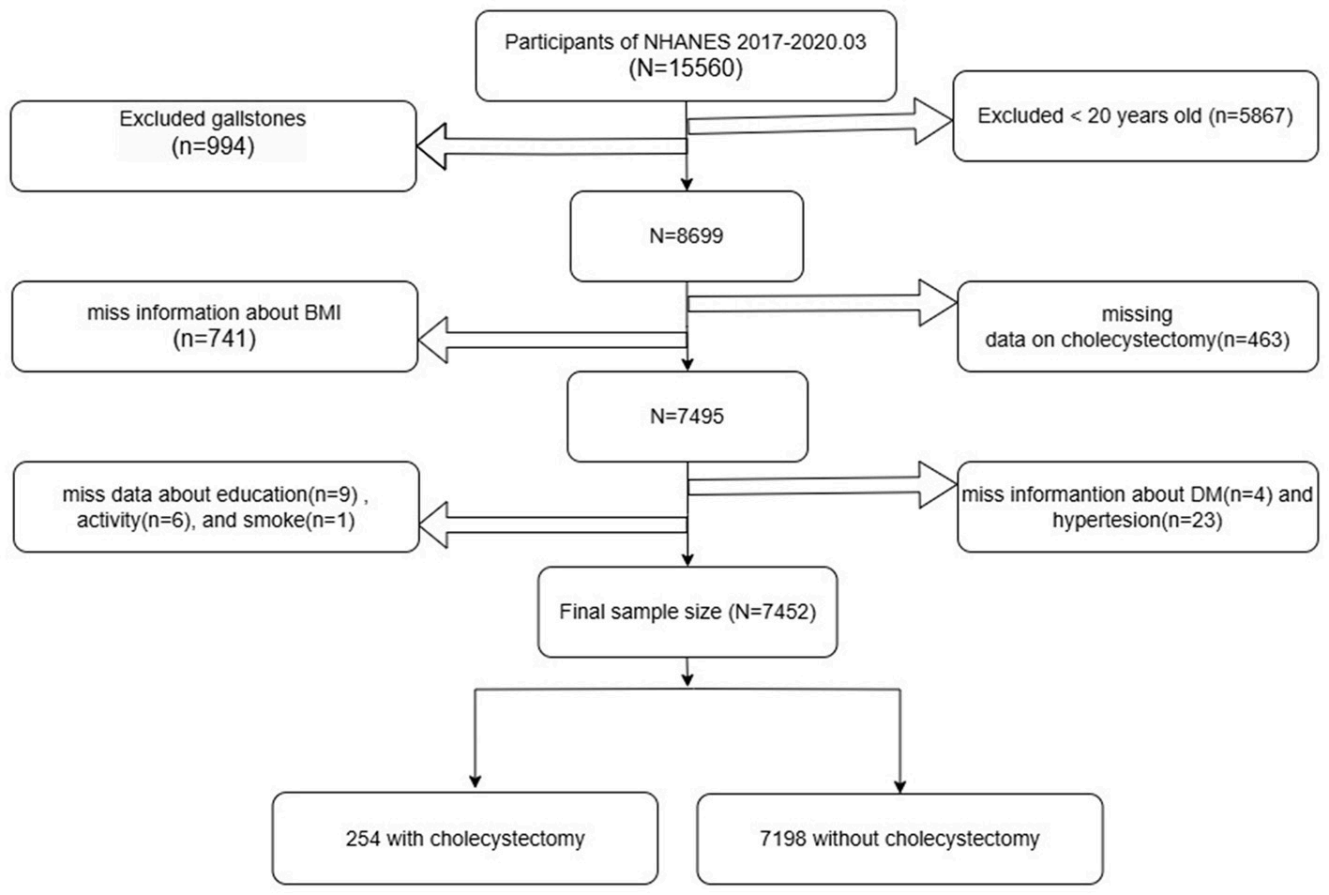
Flowchart for study population.

Baseline participation included 15,560 individuals who completed the initial assessment. Of these, 5,867 participants under 20 years of age and 994 individuals diagnosed with gallstones were excluded from the analysis. Further exclusions were made for participants with missing data on cholecystectomy status (*n* = 463) and body mass index (BMI) (*n* = 741). In addition, 43 participants lacking essential information on diabetes mellitus (*n* = 4), hypertension (*n* = 23), educational attainment (*n* = 9), work activity (*n* = 6), or smoking status (*n* = 1) were also excluded. After applying these criteria, a total of 7,452 participants remained eligible for analysis, comprising 254 individuals with a history of cholecystectomy and 7,198 without. The detailed inclusion and exclusion process is presented in Figure 1.

### Definition of gallstones and gallbladder surgery

The presence of gallstones and history of gallbladder surgery were determined through structured participant interviews using standardized questions. Specifically, participants were asked: “Has a doctor or other health professional ever told you that you had gallstones? (MCQ550)” and “Have you ever had gallbladder surgery? (MCQ560).” Individuals who answered “yes” to the first question were classified as having gallstones, while those who answered “no” were considered free of gallstones. Similarly, respondents who replied “yes” to the second question were considered to have undergone gallbladder surgery, and those who replied “no” were classified as having no such surgical history. To ensure data quality and accuracy, real-time consistency checks were implemented through Computer-Assisted Personal Interview (CAPI) systems, and post-interview audits, including audio recording reviews, were conducted by National Center for Health Statistics (NCHS) staff.

### Identification of covariates

Potential covariates for analysis were systematically identified in accordance with existing literature (8–11). This study utilized data extracted from the NHANES database, with key variables spanning several domains: ① Demographic information, such as sex, age, race, and education level (classified as below high school, high school graduate, or above high school); ② Socioeconomic status, assessed by the family poverty-to-income ratio (PIR); ③ Health conditions, specifically the presence of hypertension and diabetes; ④ Lifestyle factors, including levels of physical activity and smoking status; ⑤ Anthropometric measurements, comprising body mass index (BMI), waist and hip circumferences, and liver stiffness and Hepatic Steatosis assessed with ultrasound elastography; ⑥ and Laboratory data, including complete blood count, lipid profile, and liver function tests. Diagnoses of hypertension and diabetes were established based on a combination of self-reported questionnaire data, clinical examinations, and relevant laboratory findings. Physical activity was categorized using information from the NHANES Physical Activity Questionnaire (PAQ), while smoking status was determined based on participant responses in standardized surveys. The FibroScan^®^ machine has incorporated a novel physical parameter (controlled attenuation parameter or CAP^TM^), which measures the ultrasound attenuation related to the presence of hepatic steatosis. The CAP measurement is recorded simultaneously with the liver stiffness measurement.

### Statistical analysis

Continuous variables were summarized as mean ± standard deviation, and categorical variables were described as frequencies with corresponding percentages. Inter-group comparisons were performed using independent-sample *t*-tests or one-way ANOVA for continuous variables and Chi-square tests for categorical variables. To assess the relationship between cholecystectomy and incident BMI, individuals with the outcome at baseline were excluded. The primary analysis employed multivariable linear regression to evaluate the association between cholecystectomy and BMI, treating BMI as a continuous outcome variable. Secondary analyses included logistic regression models when clinically relevant dichotomizations of BMI were considered. Subgroup analyses were conducted to examine potential effect modification across clinically relevant strata. To minimize potential confounding, propensity score methods were applied. Propensity scores were estimated using logistic regression based on prespecified covariates, and propensity score matching or weighting (including overlap weighting) was conducted as appropriate. Causal mediation analyses were performed to assess the mediating role of hepatic steatosis—quantified by controlled attenuation parameter (CAP)—in the association between cholecystectomy and BMI outcomes. Mediation effects were assessed using R's mediation package with 5,000 bootstrap iterations for robust confidence interval estimation. Missing data were handled using multiple imputation techniques when applicable. Statistical analyses were performed using R (version 3.3.2) and Free Statistics software (version 2.1.1). Two-sided *p*-values < 0.05 were considered statistically significant.

## Results

### Baseline characteristics

Table 1 presents the baseline characteristics of the 7,452 participants. The overall mean age was 49.8 ± 17.4 years, while individuals in the cholecystectomy group had a higher mean age of 61.0 ± 15.3 years. Among participants who underwent cholecystectomy, the female-to-male ratio was 2.22:1. The cholecystectomy group also exhibited a significantly higher mean BMI (31.8 ± 8.1) compared to the non-cholecystectomy group (*p* < 0.05). Furthermore, the prevalence of hypertension, diabetes mellitus, increased waist circumference, elevated white blood cell count, and higher hs-CRP levels were all significantly greater in participants with a history of cholecystectomy (all *p* < 0.05).

**Table 1:** Baseline characteristics of participants.

**Variables**	**Total (*n =* 7,452)**	**Non-cholecystectomy (*n =* 7,198)**	**Cholecystectomy (*n =* 254)**	***P* value**
Age, Mean ± SD	49.8 ± 17.4	49.5 ± 17.4	61.0 ± 15.3	< 0.001
**Sex**, ***n*** **(%)**				
Male	3,776 (50.7)	3,697 (51.4)	79 (31.1)	< 0.001
Female	3,676 (49.3)	3,501 (48.6)	175 (68.9)	
**Race**, ***n*** **(%)**				
Non-Hispanic White	2,498 (33.5)	2,351 (32.7)	147 (57.9)	< 0.001
Non-Hispanic Black	2,048 (27.5)	2,002 (27.8)	46 (18.1)	
Mexican American	851 (11.4)	829 (11.5)	22 (8.7)	
Other race	2,055 (27.6)	2,016 (28)	39 (15.4)	
**Education level (year)**, ***n*** **(%)**				
< 9	575 (7.7)	558 (7.8)	17 (6.7)	< 0.001
9–12	2,600 (34.9)	2,479 (34.4)	121 (47.6)	
>12	4,277 (57.4)	4,161 (57.8)	116 (45.7)	
**PIR**, ***n*** **(%)**				
≤ 1.3	1,826 (28.5)	1,761 (28.4)	65 (30.1)	
>1.3, < 3.5	2,494 (38.9)	2,396 (38.7)	98 (45.4)	
≥3.5	2,095 (32.7)	2,042 (32.9)	53 (24.5)	
**Smoking status**, ***n*** **(%)**				
Never	4,386 (58.9)	4,256 (59.1)	130 (51.2)	0.021
Former	1,689 (22.7)	1,615 (22.4)	74 (29.1)	
Current	1,377 (18.5)	1,327 (18.4)	50 (19.7)	
**Moderate activity**, ***n*** **(%)**				
No	4,177 (56.1)	4,024 (55.9)	153 (60.2)	0.172
Yes	3,275 (43.9)	3,174 (44.1)	101 (39.8)	
**Hypertension**, ***n*** **(%)**				
No	5,293 (71.0)	5,166 (71.8)	127 (50)	< 0.001
Yes	2,159 (29.0)	2,032 (28.2)	127 (50)	
**DM**, ***n*** **(%)**				
No	3,053 (84.2)	2,770 (85.5)	283 (73.9)	< 0.001
Yes	571 (15.8)	471 (14.5)	100 (26.1)	
BMI (kg/m^2^)	29.6 ± 7.3	29.6 ± 7.3	31.8 ± 8.1	< 0.001
Waist circumference (cm)	100.1 ± 16.6	99.9 ± 16.5	106.4 ± 17.1	< 0.001
TC (mg/dL)	185.9 ± 39.4	186.1 ± 39.4	181.6 ± 38.4	0.075
HDL (mg/dL)	53.7 ± 15.5	53.7 ± 15.5	53.0 ± 15.3	0.465
TG (mg/dL)	108.7 ± 64.8	108.4 ± 64.0	116.1 ± 83.6	0.063
ALT (U/L)	22.3 ± 18.3	22.4 ± 18.5	20.4 ± 14.4	0.083
AST (U/L)	22.0 ± 14.1	22.0 ± 14.2	20.7 ± 10.9	0.137
STB (mg/dL)	0.5 ± 0.3	0.5 ± 0.3	0.5 ± 0.3	0.954
hs-CRP (mg/L)	2.2 (0.9, 4.0)	2.2 (0.9, 4.0)	3.2 (1.2, 5.5)	< 0.001
WBC (10^9^/L)	7.2 ± 2.3	7.1 ± 2.3	7.6 ± 3.1	< 0.001
LSM (kPa)	5.9 ± 4.6	5.9 ± 4.6	6.4 ± 4.5	0.051
CAP (dB/m)	263.6 ± 60.7	262.9 ± 60.6	282.1 ± 62.8	< 0.001

ALT, alanine aminotransferase; AST, aspartate aminotransferase; BMI, body mass index; DM, diabetes mellitus; PIR, poverty-to-income ratio; HDL, high-density lipoprotein; STB, Serum Total Bilirubin; hs-CRP, highsensitivity C-reactive protein; TC, total cholesterol; TG, triglycerides; WBC, white blood cell; LSM, Liver Stiffness Measurement; CAP, controlled attenuated parameter.

### Associations between cholecystectomy and BMI

Univariate analysis identified multiple factors significantly associated with cholecystectomy status (Table 2). Demographic characteristics including age (*p* < 0.001), sex (*p* < 0.001), and race (*p* < 0.001) showed strong associations, with non-Hispanic White participants demonstrating the highest cholecystectomy prevalence (*p* < 0.001). Several metabolic and inflammatory markers were also significantly associated, including: Hypertension (OR 2.54, 95% CI 1.98–3.27), Diabetes mellitus (OR 2.15, 95% CI 1.61–2.88), BMI (OR 1.04, 95% CI 1.02–1.05, *p* < 0.001), Controlled attenuation parameter (CAP) scores (OR 1.01, 95% CI 1.0–1.01, *p* < 0.001), Elevated inflammatory markers (WBC: *p* = 0.002; hs-CRP: *p* = 0.04).

**Table 2:** Association between covariates and cholecystectomy status.

**Variables**	**OR (95% CI)**	***P* value**	**Variables**	**OR (95% CI)**	***P* value**
Age, Mean ± SD	1.04 (1.03–1.05)	< 0.001^*^	Smoking status, *n* (%)		
Sex, *n* (%)			Never	1 (reference)	
Male	1 (reference)		Former	1.5 (1.12–2.01)	0.006^*^
Female	2.34 (1.79–3.06)	< 0.001^*^	Current	1.23 (0.89–1.72)	0.215
Race, *n* (%)			Hypertension, *n* (%)		
Non-Hispanic White	1 (reference)		No	1 (reference)	
Non-Hispanic Black	0.37 (0.26–0.51)	< 0.001^*^	Yes	2.54 (1.98–3.27)	< 0.001^*^
Mexican American	0.42 (0.27–0.67)	< 0.001^*^	DM, *n* (%)		
Other race	0.31 (0.22–0.44)	< 0.001^*^	No	1 (reference)	
Education (year), *n* (%)			Yes	2.15 (1.61–2.88)	< 0.001^*^
< 9	1(reference)		BMI (kg/m^2^)	1.04 (1.02–1.05)	< 0.001^*^
9–12	1.6 (0.96–2.68)	0.073	WC (cm)	1.02 (1.01–1.03)	< 0.001^*^
>12	0.92 (0.55–1.53)	0.736	TC (mg/dL)	1 (0.99–1)	0.075
PIR, Mean ± SD			HDL (mg/dL)	1 (0.99–1.01)	0.465
≤ 1.3	1(reference)		TG (mg/dL)	1 (1)	0.079
>1.3, < 3.5	1.11 (0.81–1.53)	0.529	ALT (U/L)	0.99 (0.98–1)	0.066
≥3.5	0.7 (0.49–1.02)	0.061	AST (U/L)	0.99 (0.97–1)	0.115
Moderate activity, *n* (%)			LSM (kPa)	1.02 (1–1.04)	0.055
No	1(reference)		CAP (dB/m)	1.01 (1–1.01)	< 0.001^*^
Yes	0.84 (0.65–1.08)	0.172	hs-CRP (mg/L)	1.01 (1–1.02)	0.04^*^
STB (mg/dL)	0.99 (0.61–1.59)	0.954	WBC (10^9^/L)	1.06 (1.02–1.1)	0.002^*^

^*^P < 0.05.

Potential confounders were identified through univariate screening (significance threshold *p* < 0.05), including demographic factors (sex, age, race), behavioral characteristics (smoking status), clinical comorbidities (hypertension, diabetes mellitus), and metabolic-inflammatory markers (CAP, WBC, and hs-CRP). Waist circumference was excluded from final models due to significant collinearity with BMI (variance inflation factor [VIF] = 5.30). Three progressively adjusted linear regression models (Table 3) were constructed to examine the cholecystectomy-BMI association: Model 1 (Demographic-adjusted model): β = 2.21 kg/m^2^ (95% confidence interval [CI]: 1.3–3.12; *p* < 0.001) (Adjusted for: Age, sex, race, smoking status). Model 2 (Clinical-adjusted model): β = 1.71 kg/m^2^ (95% CI: 0.82–2.6; *p* < 0.001) (Additional adjustments: Hypertension, diabetes mellitus). Model 3 (Fully-adjusted model): β = 0.84 kg/m^2^ (95% CI: 0.09–1.59; *p* = 0.027) (Additional adjustments: CAP, WBC, hs-CRP). Fully-adjusted model showed good fit (*R*^2^ = 0.359–0.361) and satisfied regression assumptions.

**Table 3:** Association between cholecystectomy and BMI.

**Exposure**	**Crude**		**Model 1**		**Model 2**		**Model 3**	
	β **(95%CI)**	***P***	β **(95%CI)**	***P***	β **(95%CI)**	***P***	β **(95%CI)**	***P***
Cholecystectomy			*R*^2^ = 0.041		*R*^2^ = 0.085		*R*^2^ = 0.359	
No	R0 (Ref)		R0 (Ref)		R0 (Ref)		R0 (Ref)	
Yes	2.26 (1.34–3.18)	< 0.001^*^	2.21 (1.3–3.12)	< 0.001^*^	1.71 (0.82–2.6)	< 0.001^*^	0.84 (0.09–1.59)	0.027^*^

Crude: Unadjusted. Model 1: Adjusted for sex, race, age, and smoke status. Model 2: Adjusted for sex, race, age, smoke status, hypertension, and diabetes mellitus. Model 3: Adjusted for sex, race, age, smoke status, hypertension, diabetes mellitus, CAP, WBC, and hs-CRP. ^*^P < 0.05.

### Stratified analyses based on additional variables

**Figure 2:**
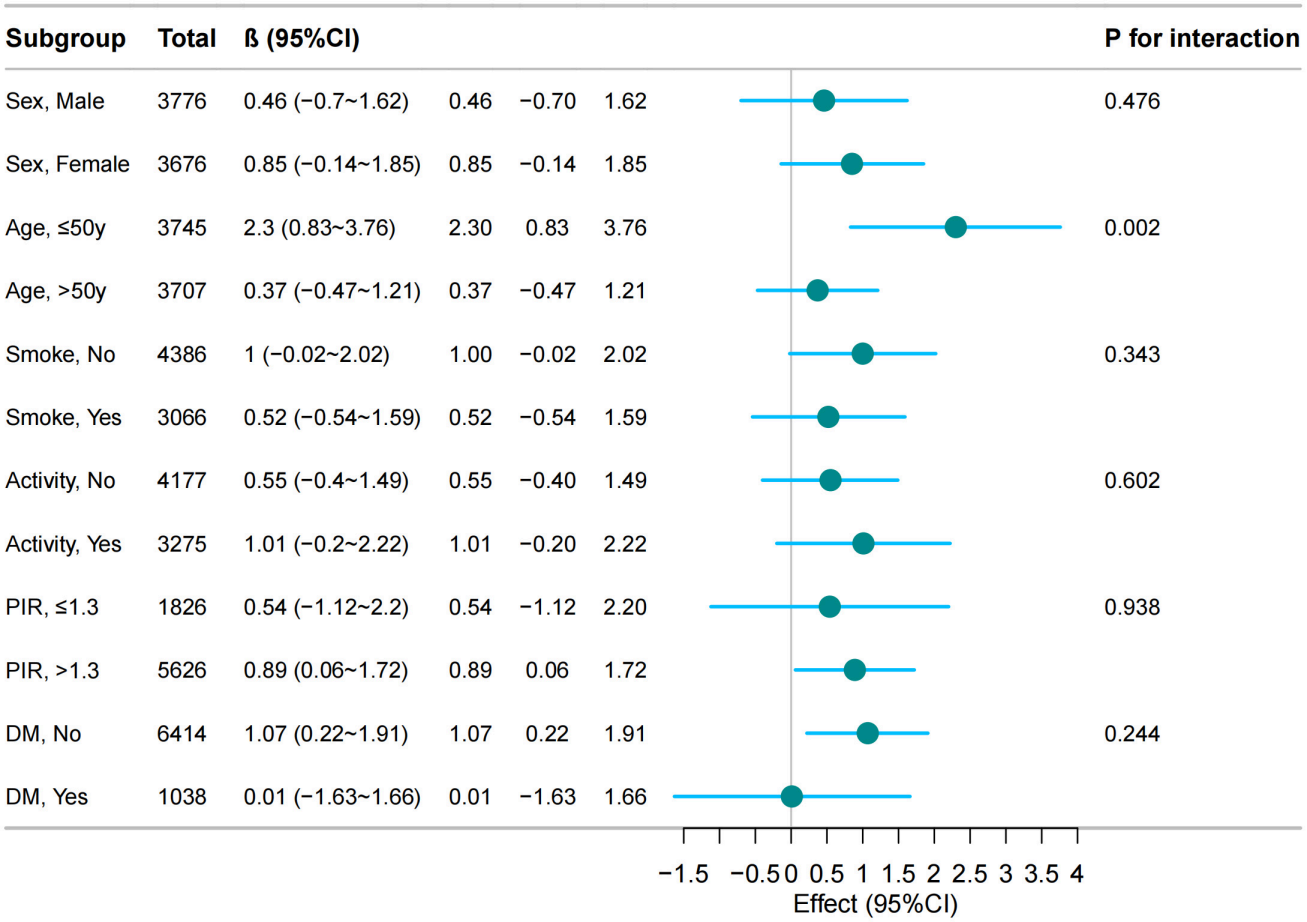
The relationship between cholecystectomy and BMI according to basic features. Except for the stratification component itself, each stratification factor was adjusted for all other variables [demographic factors (sex, age, race), behavioral characteristics (smoking status), clinical comorbidities (hypertension, diabetes mellitus), and metabolic-inflammatory markers (CAP, WBC, and hs-CRP)].

Stratified analyses revealed differential associations between cholecystectomy and BMI changes across demographic and clinical subgroups. Notably, younger patients ( ≤ 50 years) demonstrated a significant BMI increase following cholecystectomy (β = 2.3, 95% CI: 0.83–3.76, *p* = 0.002), whereas older patients (>50 years) showed no significant change (β = 0.37, 95% CI: −0.47–1.21). No significant interactions were detected for sex, socioeconomic indicators (education, poverty-income ratio), lifestyle factors (smoking, physical activity), or metabolic parameters (diabetes) (all *P* interaction > 0.05), demonstrating the robustness of the primary association across most population strata (Figure 2).

### Comparative analysis of BMI changes by postoperative duration

BMI did not differ significantly across postoperative time intervals (Table 4). In the dichotomous analysis ( ≤ 5 years vs. >5 years post-cholecystectomy), the mean BMI was 30.8 ± 7.9 kg/m^2^ (*n* = 95) and 32.1 ± 8.1 kg/m^2^ (*n* = 177), respectively (independent *t*-test, *p* = 0.202; Cohen's *d* = 0.18, indicating a small effect size). For the trichotomous grouping ( ≤ 3 years, 3.1–6 years, >6.1 years), the mean BMI values were 30.7 ± 6.0 kg/m^2^ (*n* = 50), 31.4 ± 9.4 kg/m^2^ (*n* = 52), and 32.0 ± 8.2 kg/m^2^ (*n* = 170), respectively. One-way ANOVA indicated no significant difference between groups (*F* = 0.55, *df* = 2,269; *p* = 0.578; η^2^ = 0.004, representing a minimal effect size).

**Table 4:** Comparative analysis of BMI changes across different postoperative time periods.

**Grouping method**	**Postoperative time group**	**Sample Size (*n*)**	**BMI (Mean ±SD)**	**Statistical test method**	***P*-value**	**Effect size**
Dichotomous	≤ 5 years	95	30.8 ± 7.9	Independent *t*-test	0.202	0.18 (Small)
	>5 years	177	32.1 ± 8.1			
Trichotomous	≤ 3 years	50	30.7 ± 6.0	One-way ANOVA	0.578	η^2^ = 0.004 (Minimal)
	3.1–6 years	52	31.4 ± 9.4	(*F* = 0.55, *df* = 2,269)		
	>6.1 years	170	32.0 ± 8.2			

### Propensity score analysis and model comparisons

**Figure 3:**
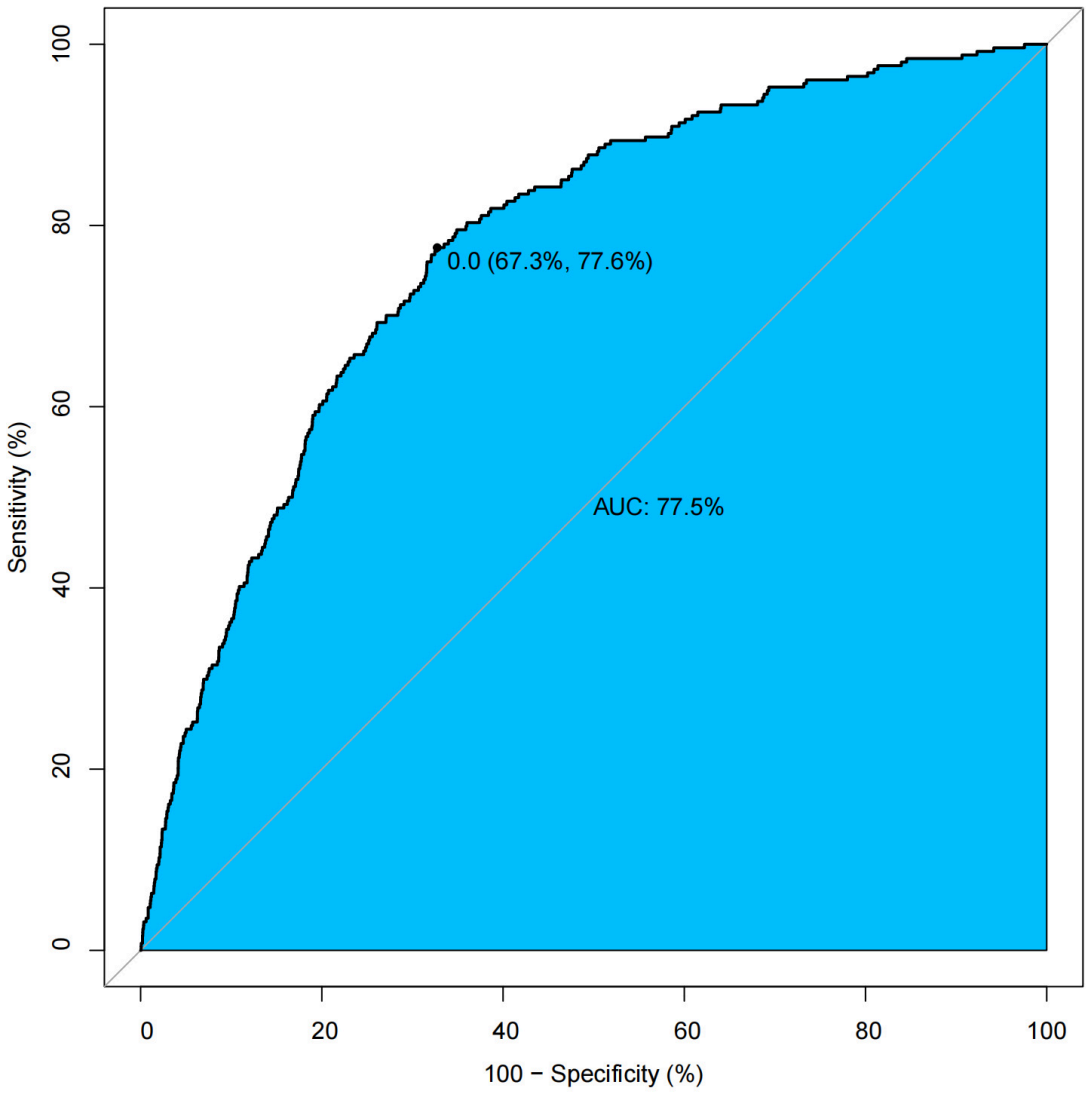
ROC Curve of the propensity score model demonstrating adequate discrimination.

The propensity score model demonstrated adequate discriminative capacity (AUC = 0.775, 95% CI: 0.673–0.776) (Figure 3). Post-matching balance diagnostics confirmed successful covariate adjustment, with all standardized mean differences (SMDs) < 0.10 across measured confounders (sex, age, race, hypertension, diabetes mellitus, CAP, white blood cell count, and CRP), indicating negligible residual imbalance (Supplementary Figure S1). The robustness of the cholecystectomy-BMI association was further validated by multiple propensity score approaches. While all methods agreed on the direction and significance of the effect, overlap weighting (Ow) yielded the most precise estimate (β = 0.91, 95% CI: 0.55–1.27, ^*^p^*^ < 0.001) (Table 5).

**Table 5:** Propensity score analysis and model comparisons.

**Models**	**β (95%CI)**	***P*-value**
Crude	2.26 (1.34–3.18)	< 0.001
Multivariable	0.79 (0.04–1.54)	0.039
PS-Adjusted	1.02 (0.09–1.94)	0.031
PS-Matched	1.36 (0.07–2.65)	0.039
IPTW	1.45 (0.52–2.39)	0.002
SMRW	0.85 (−0.1–1.81)	0.08
PA Weighting	0.85 (0.5–1.21)	< 0.001
Ow	0.91 (0.55–1.27)	< 0.001

PS, Propensity Score; IPTW, Inverse Probability of Treatment Weighting;

SMRW, Standardized Mortality Ratio Weighting; Ow, Overlap Weighting.

### Mediating role of fatty liver in the association between exposure and outcome

**Figure 4:**
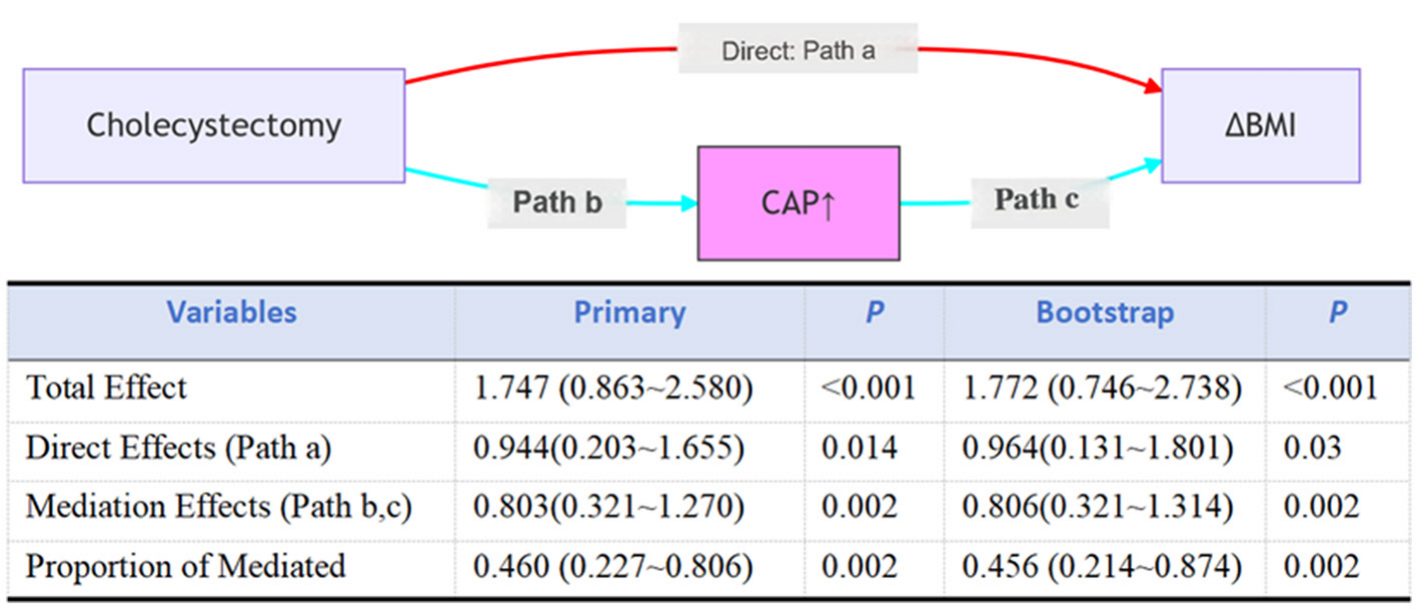
Mediation analysis for hepatic steatosis, as quantified by controlled attenuation parameter (CAP), in the association between cholecystectomy and BMI. Total effect: the overall impact of cholecystectomy (X) on BMI (Y), without considering the mediating effect of CAP (M); Direct effect: the direct impact of cholecystectomy (X) on BMI (Y) after controlling for the effect of CAP (M); Indirect effect: the indirect impact of cholecystectomy (X) on BMI (Y) through CAP (M); Percent mediation: the proportion of the indirect effect in the total effect, reflecting the importance of CAP (M) in the relationship between cholecystectomy and BMI. Adjusted for age, sex, race, smoke status, diabetes status, and white blood cell count.

Causal mediation analysis (Figure 4) revealed that hepatic steatosis, as quantified by controlled attenuation parameter (CAP), significantly mediated the exposure-outcome association after adjusting for covariates (age, sex, race, smoke status, diabetes status, and white blood cell count). The mediated proportion reached 46.0% (95% CI: 22.7%−80.6%) in primary analysis vs. 45.6% (21.4%−87.4%) in bootstrap validation, with an average causal mediation effect (ACME) of 0.803 kg/m^2^ (95% CI: 0.321–1.270; *P* = 0.002) vs. 0.806 kg/m^2^ (95% CI: 0.321–1.314; *P* = 0.002) in bootstrap validation. The exposure maintained a significant direct effect (ADE = 0.964, 95% CI: 0.131–1.801; *p* = 0.03), with a total effect size of 1.772 (95% CI: 0.746–2.738; *p* < 0.001) in bootstrap validation.

## Discussion

In this cross-sectional study, we investigated the relationship between cholecystectomy and BMI. Our findings demonstrate that cholecystectomy is an independent risk factor for increased BMI, particularly in young and middle-aged adults, with hepatic steatosis mediating nearly half of this effect (46%). These findings challenge the prevailing notion that post-cholecystectomy weight gain is attributable solely to dietary changes (4, 12, 13), and instead indicate a direct metabolic consequence of gallbladder removal (8).

Evidence from a cross-sectional study in China revealed a significantly higher prevalence of metabolic syndrome (MetS) among patients with a history of cholecystectomy (63.5%), compared with those with gallstones (47.0%) or without gallstone disease (30.3%) (14). However, the cross-sectional design limited causal inference. In contrast, a large-scale longitudinal cohort study from South Korea (9) the cross-sectional design limited causal inference. It is important to interpret previous results with caution, as the diagnosis of MetS relies on the presence of at least three out of five possible criteria (15). This composite nature introduces heterogeneity and potential confounding due to varying combinations of diagnostic components (16). To mitigate this limitation, our study focused on assessing the association between cholecystectomy and BMI, an objectively measured continuous variable, rather than using MetS as the primary endpoint. This design enhances the precision of our findings by minimizing diagnostic variability and reducing confounding from the differential weighting of MetS components. Supporting this approach, Chen et al. (17) observed an increased risk of GSD with a greater number of MetS components. To further isolate the effect of gallbladder removal, we excluded participants with pre-existing GSD from our analysis.

Mechanistically, cholecystectomy disrupts the physiological pattern of bile acid secretion, leading to altered signaling through FXR, GPBAR-1, and FGF19 in multiple tissues (18). These molecular changes are implicated in insulin resistance, the development of hepatic steatosis, and dysregulated lipid profiles (19). Importantly, our findings quantify the mediating role of fatty liver, which explains approximately 46% of the pathway from cholecystectomy to increased BMI. This result highlights fatty liver as a key intermediary, not merely a concurrent condition. However, as with any cross-sectional mediation analysis, this estimate should be interpreted with caution, as it could be influenced by pre-existing metabolic conditions that may contribute to both hepatic steatosis and the need for cholecystectomy. Additionally, changes in the gut microbiota following cholecystectomy may further contribute to metabolic dysregulation (20). Subgroup analysis in our study revealed a more pronounced effect in younger individuals ( ≤ 50 years). This greater vulnerability may be due to higher metabolic plasticity and less mature compensatory mechanisms in younger adults, making them more sensitive to disruptions in biliary physiology. Age-related differences in bile acid processing have also been observed, with young rat livers showing increased biotransformation compared to adults (21). In contrast, older patients often experience more postoperative complications, which may reflect different underlying pathophysiological processes (22, 23). Clinically, these findings underscore the importance of postoperative metabolic monitoring and lifestyle counseling, especially for younger patients undergoing cholecystectomy.

Although the observed mean BMI differences were modest, they may carry meaningful clinical implications at the population level, as a 1–2 kg/m^2^ increase is associated with a 15–20% higher risk of metabolic syndrome and a 5–10% increased cardiovascular disease risk (24). These effects could be especially pronounced in younger patients ( ≤ 50 years), who showed a greater BMI increase of 2.3 kg/m^2^—comparable to weight gain typically occurring over 5–8 years in midlife. The lack of significant association between postoperative intervals and BMI changes should be interpreted cautiously, primarily due to recall bias from self-reported surgical history. Inaccurate recollection of surgery dates may misclassify exposure timing and dilute measurable effects, likely contributing to the null findings.

Cholecystectomy is an effective treatment with low rates of mortality and morbidity (2, 25). Studies consistently show that while elective cholecystectomy carries relatively low risk in elderly patients, mortality rates remain notably higher than zero, particularly in those over 70 years (26, 27). However, cholecystectomy may contribute to long-term metabolic complications by altering digestive physiopathology. Evidence indicates that cholecystectomy can disrupt the enterohepatic circulation of bile acids, which may in turn affect glucose, insulin, and lipid metabolism (9, 19). This disruption can also lead to gut dysbiosis, manifesting as reduced microbial diversity and an increased abundance of pro-inflammatory bacteria (28). After cholecystectomy, bile acids flow continuously into the intestine, disrupting enterohepatic cycling and promoting dysbiosis. This includes reduced diversity, depletion of beneficial short-chain fatty acid-producing bacteria, and expansion of pro-inflammatory taxa (19, 28). Bile acids act as signaling molecules through receptors such as FXR and TGR5, which regulate glucose, lipid, and energy homeostasis (5). Interestingly, FXR-deficient mice on a methionine- and choline-deficient diet showed less hepatic steatosis but increased inflammation and fibrosis compared to wild-type mice, possibly due to elevated hepatic bile acid levels inhibiting fatty acid uptake and triglyceride accumulation (29). Thus, the gallbladder serves as a physiological regulator of bile acid metabolism, contracting rhythmically in response to meals to maintain metabolic homeostasis (30, 31). Together, these findings underscore the integral role of the gallbladder in metabolic regulation through bile acid-mediated pathways.

In summary, this cross-sectional study suggests an association between cholecystectomy and increased BMI, with hepatic steatosis appearing to mediate a substantial portion of this relationship, particularly in younger adults. These findings highlight the need for further investigation into the potential metabolic consequences of cholecystectomy and suggest that clinicians might consider metabolic monitoring in post-operative care, especially for younger patients.

## Limitations

This study has several important limitations. First, the cross-sectional design fundamentally limits causal inference, as temporal sequence between cholecystectomy and BMI change cannot be established. Second, although mediation analysis suggested a significant role of hepatic steatosis, unmeasured confounding—particularly by pre-existing metabolic conditions—threatens interpretability. Specifically, baseline steatosis may have contributed to both gallstone formation (leading to surgery) and subsequent BMI increase, potentially inflating the estimated mediation effect. Third, selection bias may arise from missing preoperative metabolic profiles and exclusion of participants with incomplete data. Finally, measurement error from self-reported surgery history and unaccounted lifestyle changes post-surgery may further bias effect estimates. While propensity score and multiple imputation methods were employed, residual confounding remains plausible.

## Data Availability

The datasets presented in this study can be found in online repositories. The names of the repository/repositories and accession number(s) can be found in the article/Supplementary material.

## Supplementary material

Supplementary Figure S1 Standardized mean differences of covariates before and after matching.
