# Supplementary figures and images for: Hepatic steatosis mediates the relationship between cholecystectomy and BMI increase: a population-based study
Source: Front Med (Lausanne). 2025 Sep 30;12:1620036. doi: 10.3389/fmed.2025.1620036 (PMC12518335; doi:10.3389/fmed.2025.1620036)

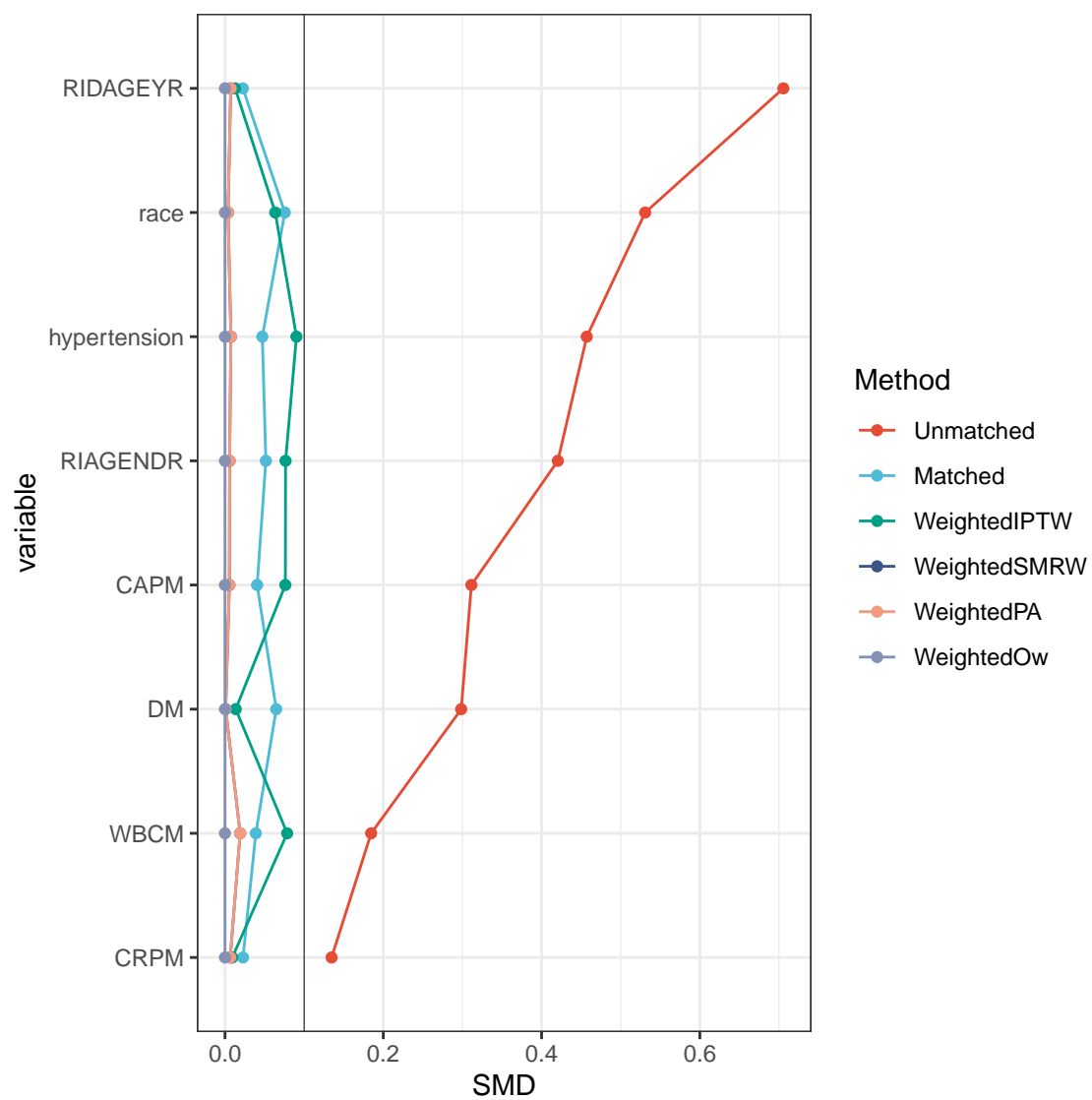

Supplement: Supplementary Figure S1 — Standardized mean differences of covariates before and after matching. [file Data_Sheet_1.pdf]
